# A gene expression profile-based approach to screen the occurrence and predisposed host characteristics of drug-induced liver injury: a case study of *Psoralea corylifolia* Linn

**DOI:** 10.3389/fchem.2023.1259569

**Published:** 2023-10-06

**Authors:** Ming-Liang Zhang, Wei-Xia Li, Xiao-Yan Wang, Hui Zhang, Ya-Li Wu, Liu-Qing Yang, Xiao-Fei Chen, Shu-Qi Zhang, Yu-Long Chen, Ke-Ran Feng, Jin-Fa Tang

**Affiliations:** ^1^ The Department of Pharmacy, First Affiliated Hospital of Henan University of Traditional Chinese Medicine, Zhengzhou, China; ^2^ Henan Province Engineering Research Center for Clinical Application, Evaluation and Transformation of Traditional Chinese Medicine, Zhengzhou, China; ^3^ Henan Provincial Key Laboratory for Clinical Pharmacy of Traditional Chinese Medicine, Zhengzhou, China; ^4^ Henan Province Engineering Research Center of Safety Evaluation and Risk Management of Traditional Chinese Medicine, Zhengzhou, China; ^5^ Henan University of Traditional Chinese Medicine, Zhengzhou, China

**Keywords:** drug-induced liver injury, differentially expressed genes, predict, host characteristics, psoralen

## Abstract

Drug-induced liver injury (DILI) is one of the most common causes of a drug being withdrawn, and identifying the culprit drugs and the host factors at risk of causing DILI has become a current challenge. Recent studies have found that immune status plays a considerable role in the development of DILI. In this study, DILI-related differentially expressed genes mediated by immunoinflammatory cytokines were obtained from the Gene Expression Omnibus (GEO) database to predict the occurrence of DILI (named the DILI predictive gene set, DILI_PGS), and the predictability of the DILI_PGS was verified using the Connectivity Map (CMap) and LiverTox platforms. The results obtained DILI_PGS from the GEO database could predict 81.25% of liver injury drugs. In addition, the Coexpedia platform was used to predict the DILI_PGS-related characteristics of common host diseases and found that the DILI_PGS mainly involved immune-related diseases and tumor-related diseases. Then, animal models of immune stress (IS) and immunosuppressive (IP) were selected to simulate the immune status of the above diseases. Meanwhile, psoralen, a main component derived from *Psoralea corylifolia* Linn. with definite hepatotoxicity, was selected as an experimental drug with highly similar molecular fingerprints to three idiosyncratic hepatotoxic drugs (nefazodone, trovafloxacin, and nimesulide) from the same DILI_PGS dataset. The animal experiment results found a single administration of psoralen could significantly induce liver injury in IS mice, while there was no obvious liver function change in IP mice by repeatedly administering the same dose of psoralen, and the potential mechanism of psoralen-induced liver injury in IS mice may be related to regulating the expression of the TNF-related pathway. In conclusion, this study constructed the DILI_PGS with high accuracy to predict the occurrence of DILI and preliminarily identified the characteristics of host factors inducing DILI.

## 1 Introduction

Drug-induced liver injury (DILI) is a common cause of drug withdrawal, restriction, and termination of marketing (Regev, 2014). It is a rare but potentially severe adverse drug event with serious clinical results, including acute liver failure and the need for liver transplantation (European Association for the Study of the Liver, 2019), and acute DILI accounts for more than 50% of all cases of acute liver failure (Reuben et al., 2016; Donnelly et al., 2017). The elimination of iatrogenic “injury” caused by treatment purposes is a priority of patient care. In this regard, the websites of LiverTox (https://www.ncbi.nlm.nih.go) and DILIrank (https://www.fda.gov/science-research/liver-toxicity-knowledge-base-ltkb/drug-induced-liver-injury-rank-dilirank-dataset) have included more than 1,200 kinds of chemical drugs and dietary supplements that can induce liver injury, providing a certain reference basis for avoiding the occurrence of DILI (Hoofnagle et al., 2013; Chen et al., 2016).

However, DILI is typically classified as either intrinsic or idiosyncratic. In contrast to intrinsic DILI, in which the damage is dose-dependent and occurs transitorily (hours to days) after exposure to the drug with the occurrence mechanism and prevention-control methods clear and predictable, idiosyncratic DILI (IDILI), which occurs without obvious dose dependence and with long latency (weeks to months), is multifactorial and unpredictable (European Association for the Study of the Liver, 2019). Recently, indirect DILI has been described as a third type of DILI caused by the mechanism of action of the drug due to its idiosyncratic properties (Hoofnagle and Björnsson, 2019). At present, it is generally believed that the occurrence of IDILI is closely related to host factors (e.g., genetics and immunity) (Chalasani et al., 2021). Although the occurrence of IDILI is relatively rare, once it occurs, about 10% of patients may experience life-threatening clinical outcomes, such as acute liver failure, with most requiring a liver transplant or ultimately mortality; if not recognized and the drug causing it is not discontinued promptly, some patients will develop chronic DILI (Wei et al., 2007; Reuben et al., 2010; Chalasani et al., 2015). Therefore, identifying the host factors that cause individual susceptibility is the focus and difficulty of the ongoing DILI research.

Some key features of IDILI drugs that induce liver injury are mediated by the adaptive immune system, and almost all forms of idiosyncratic drug-induced hepatitis exhibit features of drug hypersensitivities, such as rash, fever, and eosinophilia (Devarbhavi et al., 2011; Chalasani et al., 2015). Drug-protein adducts formed from drugs or secondary metabolites interacting with host proteins as haptens are generally considered to be presented as neoantigens by the major histocompatibility complex Class II, playing a key role in adaptive immune responses (Hoofnagle and Björnsson, 2019). Studies have shown that the inflammatory microenvironment can enhance the specificity of DILI (Jiang et al., 2017). Thus, the gene spectrum composed of a series of related differentially expressed genes (DEGs) that enhance the specificity of DILI in the inflammatory microenvironment may partially characterize the organism status of susceptible individuals at risk of IDILI.

Connectivity Map (CMap) (https://clue.io/query) is a database platform that contains more than 5,000 marketed drugs or small molecule compounds with potential pharmacological activity that interfere with the expression of perturbed genes in 80 human cell lines, and provides a method to quantify the disease-drug similarity “link score” criteria and online analysis tools based on pattern matching algorithms (Subramanian et al., 2017). Over the past 15 years, drug repurposing and disease redefining conducted based on the database has provided biomedical insights and treatment clues for a variety of diseases, including coronavirus disease 2019 (COVID-19) (Chen et al., 2021; Li et al., 2022). The Coexpedia platform (https://www.coexpedia.org/) is a data mining system that integrates functional hypotheses extracted from transcriptomic data sources in the Gene Expression Omnibus (GEO) database (https://www.ncbi.nlm.nih.gov/geo/) and provides a new approach for human disease research based on gene expression profile similarity (Yang et al., 2017). Therefore, it may be feasible to first obtain the DILI-related DEG profile induced by inflammatory microenvironments based on the GEO database, repurpose small molecular compounds with similar gene expression profiles for DILI-related DEGs according to the CMap database, verify the prediction ability of the DILI-related DEGs profiles to characterize hepatotoxicity by querying the LiverTox database, and further mine and analyze the characteristics of potential susceptible diseases inducing DILI based on the Coexpedia database.

**FIGURE 1:**
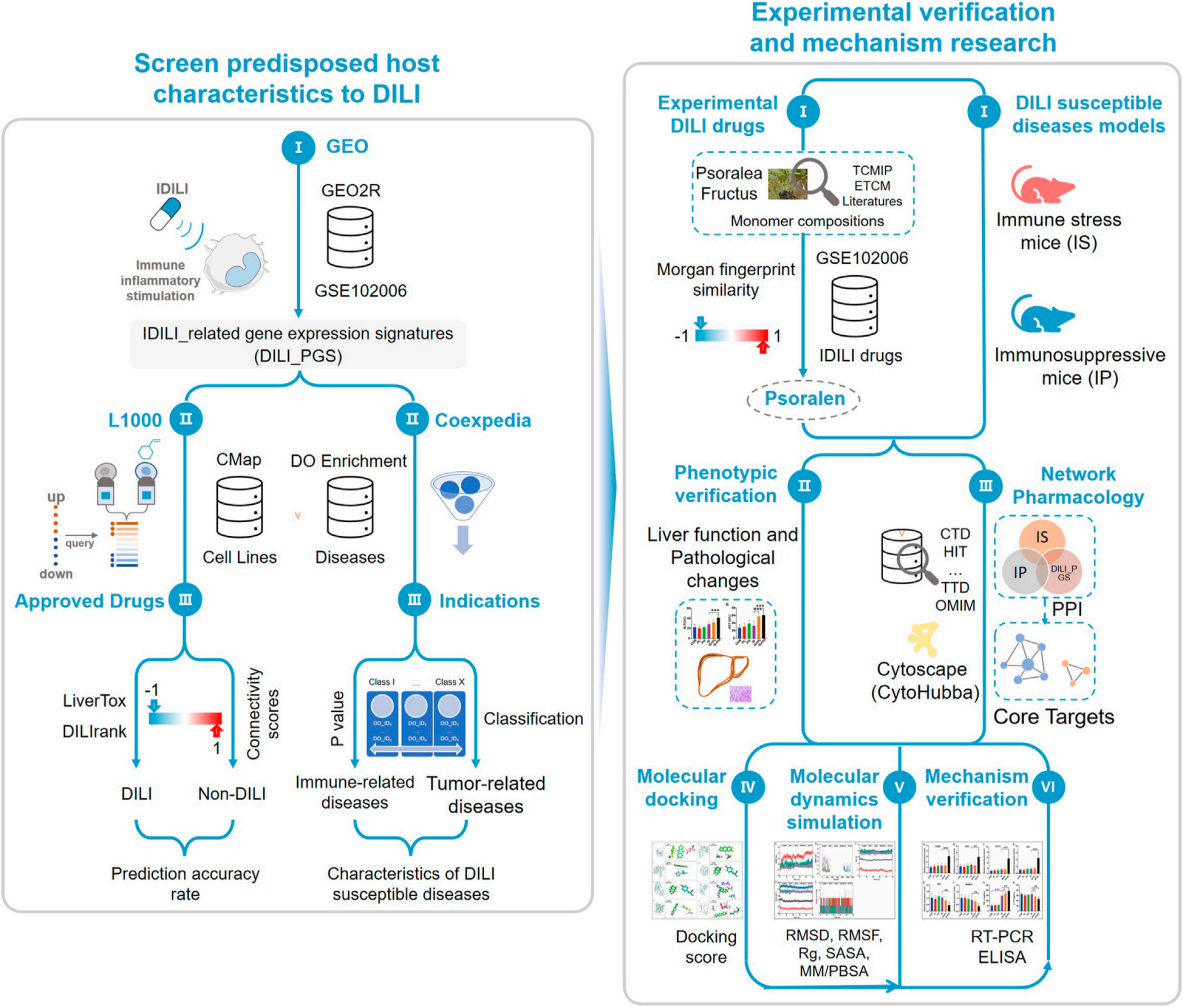
Study schematic diagram. By integrating databases of GEO, CMap, LiverTox, DILIrank, and Coexpedia, the study obtained drug-induced liver injury (DILI)-related gene expression signatures with certain accuracy and the host characteristics predisposed to DILI. According to the Morgan molecular fingerprint similarity algorithm, the monomer components with high molecular fingerprint similarity of the idiosyncratic drugs in GSE102006 were selected from the known hepatotoxic traditional Chinese medicine *Psoralea corylifolia* Linn. (BGZ) as the experimental drugs and the host status of DILI susceptibility was characterized by the immune stress (IS) animal model and the immunosuppressive (IP) animal model, then phenotypic verification was carried out using the above experimental drugs and animal models. Network pharmacology, molecular docking, and molecular dynamics simulation were used to preliminarily screen the core target, and reverse transcription-polymerase chain reaction (RT-PCR) and enzyme-linked immunosorbent assay (ELISA) were used to verify the above core targets.

*Psoralea corylifolia* Linn. (BGZ), a dried and mature fruit of Psoralea corylifolia L. from a leguminous plant, has been reported to have hepatotoxicity in recent years (Wang et al., 2020a; Ge et al., 2021), but its hepatotoxic components (including bakuchiol and psoralen) are still controversial (Park et al., 2005; Li et al., 2017; Jiang et al., 2022; Ma et al., 2022). Morgan fingerprints are a method of encoding molecular structure and are a highly informative representation for many chemical analog predictions via extended connectivity fingerprinting (Rogers and Hahn, 2010; Chang et al., 2018). Accordingly, the study plans to clarify the feasibility of the DILI-related DEGs profiles to predict the characteristics of DILI and preliminarily identify the hepatotoxic components of BGZ (Figure 1).

## 2 Materials and methods

### 2.1 Construct a gene set to predict the occurrence of DILI

#### 2.1.1 Acquisition of the DILI-related DEGs

The gene expression data of DILI were acquired from the GEO database by searching the terms “idiosyncratic drug-induced liver injury”. The collected datasets were further selected if they met the following inclusion criteria: (a) whole-genome transcriptome profiling, and (b) species of origin were “*Homo sapiens*”. DEGs with an adjusted *p* value <0.05 and ǀLog_2_ fold changeǀ≥1 were identified as the DILI predictive gene set (DILI_PGS).

#### 2.1.2 Prediction of DILI-associated drugs by the DILI_PGS

The composition characteristics of the DILI_PGS, including significantly up- and downregulated DEGs, were used as the input data and compared with the gene expression signatures of available CMap drugs by using a pattern-matching algorithm based on the non-parametric rank-ordered Kolmogorov–Smirnov statistic to determine connectivity scores (Subramanian et al., 2017) and obtain the drugs with similar gene expression profiles to the DILI_PGS. The results of signature matching for each pair are shown with connectivity scores (CS) ranging from −1 to +1 (Xing et al., 2022). The closer the CS is to 1, the stronger the positive correlation, and the closer the CS is to −1, the stronger the negative correlation. Among them, drugs with a significant positive CS have the potential to induce DILI, and drugs with a significant negative CS have the potential to reverse DILI.

#### 2.1.3 Feasibility evaluation of the DILI_PGS

The drugs acquired by the CMap platform were further filtered according to |CS)| ≥ 0.60 and inclusion in the Drugbank database (https://go.drugbank.com/), and the percentage of hepatotoxicity drugs was calculated based on the databases containing hepatotoxic drugs (specifications of origin were “*Homo sapiens*”, excluding cell level), such as LiverTox and DILIrank, to evaluate the feasibility of predicting the host characteristics of patients with DILI based on the DILI_PGS.

### 2.2 Analysis of disease characteristics inducing DILI

#### 2.2.1 Analysis of the immune cell infiltration for the corresponding DILI_PGS

Cell-type Identification By Estimating Relative Subsets Of RNA Transcripts (CIBERSORT) (https://cibersort.stanford.edu/) was used to comprehensively estimate the infiltration level of each immune cell subtype between the IDILI with inflammatory factor stimulation (IDILI_with) and IDILI without inflammatory factor stimulation (IDILI_without) based on linear support vector regression (Newman et al., 2015). Estimation of STromal and Immune cells in MAlignant Tumours using Expression data (ESTIMATE) (https://bioinformatics.mdanderson.org/estimate/) was used to assess the immune scores, stromal scores, estimate scores, and tumor purity between the IDILI_with group and IDILI_without group (Yoshihara et al., 2013). The analyses of CIBERSORT and ESTIMATE were completed using the Sangerbox tool (http://sangerbox.com/home.html) (Shen et al., 2022).

#### 2.2.2 Gene set enrichment analysis

The study used the Gene Set Enrichment Analysis (GSEA) software (version 3.0) to assess the differentially enriched pathways between the IDILI_with group and IDILI_without group according to inflammatory factor stimulation conditions, and the gene set of ‘c2.cp.kegg.v7.4.symbols.gmt’ was downloaded to evaluate the Kyoto Encyclopedia of Genes and Genomes (KEGG) pathways from the Molecular Signatures Database (http://www.gsea-msigdb.org/gsea/downloads.jsp). The minimum gene set was five, the maximum gene set was 5,000, and 1,000 resampling, *p* < 0.05, and false discovery rate (FDR) < 0.25 were considered statistically significant (Subramanian et al., 2005). The analysis of GSEA was completed using the Sangerbox tool (Shen et al., 2022).

#### 2.2.3 Analysis of disease characteristics of the corresponding gene set associated with DILI

The DILI_PGS was analyzed using the COEXPEDIA platform for disease ontology (DO) analysis to explore the disease characteristics that potentially induce DILI, which is a database of context-associated co-expression networks inferred from individual series’ of microarray samples for human and mouse samples based on the GEO database (Yang et al., 2017).

### 2.3 Molecular fingerprint similarity calculation

In the study, the similarity of molecular fingerprints between the related components of BGZ and IDILI-related drugs acquired from the data sets of the GEO database was calculated by Morgan fingerprints. The related components of BGZ were obtained from Traditional Chinese Medicine Systems Pharmacology (TCMSP) (https://tcmsp-e.com/tcmsp.php), Encyclopedia of Traditional Chinese Medicine (ETCM) (http://www.tcmip.cn/ETCM/index.php/Home/Index/), Pubmed (https://pubmed.ncbi.nlm.nih.gov/), and other databases, and the Structure Data File (SDF) structures of BGZ-related monomer compounds and IDILI-related drugs were downloaded from the PubChem database (https://pubchem.ncbi.nlm.nih.gov/). Morgan fingerprints were acquired by RDKit (Rogers and Hahn, 2010), and the similarity coefficient ranges from 0 to 1; the larger the value, the higher the similarity. Ultimately, BGZ-related compounds with high similarity were selected for subsequent experimental verification.

### 2.4 Animal experimental verification

#### 2.4.1 Establishment of animal models and treatment

C57BL/6 female mice, 6–8 weeks of age (16–18 g), were obtained from SPF Biotechnology Co., Ltd. (License No. SCXK20190010, Beijing, China) and housed in the Laboratory Animal Center of the Fifth Medical Center, Chinese PLA General Hospital (Animal Ethics Committee approval No. YFYDW2020017). All mice were raised under specific pathogen-free conditions under a 12 h light/dark cycle, with free access to adequate food and water. All animals were fed adaptively for 1 week before starting the experiments. The mice were randomly divided into the normal group (CON), low-dose psoralen group (PL, 40 mg/kg), high-dose psoralen group (PH, 80 mg/kg), immunological stress group (IS), immunological stress-treated with PL group (IS+PL), immunological stress-treated with PH group (IS+PH), immunosuppressive group (IP), IP-treated with PL group (IP+PL), and IP-treated with PH group (IP+PH). The number of mice in each group was eight.

For the IS model: the mice were administered via tail vein (i.v.) lipopolysaccharide (LPS) (from *Escherichia coli* O55:B5, Sigma) (2 mg/kg) in sterile saline (Wang et al., 2020b), and, 2 h later, PL, PH or its vehicle (0.5% carboxymethylcellulose sodium, 0.5% CMC-Na) was administered through gastric irrigation, respectively. Mice serum was collected 6 h after PL or PH treatment. For the IP model, the mice were administered intraperitoneal (i.p.) cyclophosphamide (CTX) in sterile saline (80 mg/kg) on days 1, 3, and 5 (Li et al., 2021a), and, from the 8th day, IP mice and normal mice were given PL, PH or its vehicle (0.5% CMC-Na) by gastric irrigation for 7 consecutive days, respectively.

#### 2.4.2 Hematoxylin and eosin staining

The left hepatic lobe was fixed with 4% paraformaldehyde for 48 h, embedded in wax, and sectioned at approximately 5 µm for hematoxylin and eosin (H&E) pathological staining analysis.

#### 2.4.3 Alanine aminotransferase and aspartate transaminase

Serum alanine aminotransferase (ALT) and aspartate transaminase (AST) levels were measured according to the manufacturer’s directions (both were purchased from Nanjing Jiancheng Bioengineering Institute, Nanjing, China).

### 2.5 Mechanism exploration and verification

#### 2.5.1 Protein–protein interaction network analysis for the DILI_PGS

The DILI_PGS was imported into the STRING database (https://cn.string-db.org/) for protein–protein interaction (PPI) network analysis, in which the organization was set as human, and an interaction score of more than 0.40 was set as the threshold. The data obtained from the STRING database were imported into Cytoscape (V3.8.0) for network visualization, and the hub targets were screened by eleven topological analysis methods of CytoHubba.

#### 2.5.2 Molecular docking

To analyze the binding affinity between ligands from BGZ and hub target proteins, Maestro software (V11.5) was used to carry out the docking scores. The structure of the ligand was downloaded from the PubChem database, and the protein structures were retrieved from the Research Collaboratory for Structural Bioinformatics (RCSB) (https://www.pdbus.org/) and Uniprot databases (https://www.uniprot.org/). For docking analysis, the ligand and proteins were optimized by the LigPrep tool and Protein Preparation Wizard, respectively. In addition, partial atomic charge attribution, protonation states generation at pH 7 ± 2.0, and energy minimization were achieved using the OPLS-2005 force field. To test the docking parameters, the ligand was docked into the catalytic pocket of the core target proteins using Grid-Based Ligand Docking with Energetics (Glide v11.5, Schrödinger) in ‘extra precision’ mode without applying any constraints.

#### 2.5.3 Molecular dynamics simulations

To verify the interactions and stability between the core target proteins and ligands, explicit solvent molecular dynamics (MD) simulations were carried out with GROMACS 2020.3 software. The simulation box size was optimized with the distance between each atom of the protein and the box greater than 1.0 nm. Filling the box with an explicit solvent-simple point chargemodel (SPC216 water molecules) and replacing the water molecules with Na* and Cl counterions to make the simulation system electrically neutral. Following the steepest descent method, energy optimization of 5.0×10^4^ steps was performed to minimize the energy consumption of the entire system and finally to reduce the unreasonable contact or atom overlap in the entire system. After energy minimization, first-phase equilibration was performed with the Canonical ensemble (NVT) ensemble at 300 K for 100 ps to stabilize the temperature of the system. Second-phase equilibration was simulated with the Constant-pressure and Constant-temperature (NPT) ensemble at 1 bar and 100 ps. The primary objective of the simulation was to optimize the interaction between the target proteins and the solvent and ions so that the simulation system was fully pre-equilibrated. All MD simulations were performed for 50 ns. PyMol2.5 was used to visualize the composite PDB format file.

#### 2.5.4 Reverse transcription-polymerase chain reaction (RT-PCR)

According to the manufacturer’s instructions, the total ribonucleic acid (RNA) in the liver was isolated by RNA-Quick Purification Kit (ES Science, RN001, China) and then synthesized into cDNA by reverse transcriptase (RT) (ES Science, RT001, China). Furthermore, cDNA amplification with appropriate primers to amplification (Table 1) was performed using Super SYBR Green qPCR Master Mix (ES Science, QP002, China) by QuantStudio 6 Flex PCR System (Applied Biosystems, USA). The cycle threshold (Ct) values of indoleamine 2,3-dioxygenase 1 (IDO1), Janus kinase 2 (JAK2), intercellular adhesion molecule 1 (ICAM1), nuclear factor kappa-B inhibitor alpha (NFKBIA), interferon regulatory factor 1 (IRF1), and signal transducer and activator of transcription 1 (STAT1) were obtained and normalized to that of Glyceraldehyde 3-phosphate dehydrogenase (GAPDH) (all reagents were from TIANYI HUIYUAN, Beijing, China), and the results were analyzed by the 2^−ΔΔCT^ method.

**TABLE 1:** Primer sequences of target genes.

Gene	Forward sequence (5’ - 3′)	Reverse sequence (5’ - 3′)
IDO1	GCT​TTG​CTC​TAC​CAC​ATC​CAC	CAG​GCG​CTG​TAA​CCT​GTG​T
JAK2	TTG​TGG​TAT​TAC​GCC​TGT​GTA​TC	ATG​CCT​GGT​TGA​CTC​GTC​TAT
ICAM1	GTG​ATG​CTC​AGG​TAT​CCA​TCC​A	CAC​AGT​TCT​CAA​AGC​ACA​GCG
NFKB1A	TGA​AGG​ACG​AGG​AGT​ACG​AGC	TTC​GTG​GAT​GAT​TGC​CAA​GTG
IRF1	ATG​CCA​ATC​ACT​CGA​ATG​CG	TTG​TAT​CGG​CCT​GTG​TGA​ATG
STAT1	TCA​CAG​TGG​TTC​GAG​CTT​CAG	GCA​AAC​GAG​ACA​TCA​TAG​GCA
GAPDH	AGG​TCG​GTG​TGA​ACG​GAT​TTG	TGT​AGA​CCA​TGT​AGT​TGA​GGT​CA

#### 2.5.5 Enzyme-linked immunosorbent assay

Serums of tumor necrosis-α (TNF-α) and interleukin 10 (IL-10) were detected using enzyme-linked immunosorbent assay (ELISA) according to the manufacturer’s directions (both were purchased from Mlbio, Shanghai, China).

### 2.6 Statistical analyses

All data are presented as mean ± SD and analyzed by GraphPad version 8. Multiple comparisons performed using ANOVA were used to analyze the liver function and core target detection by RT-qPCR or ELISA. *p* < 0.05 was considered statistically significant.

## 3 Results

### 3.1 DILI-related gene sets

**FIGURE 2:**
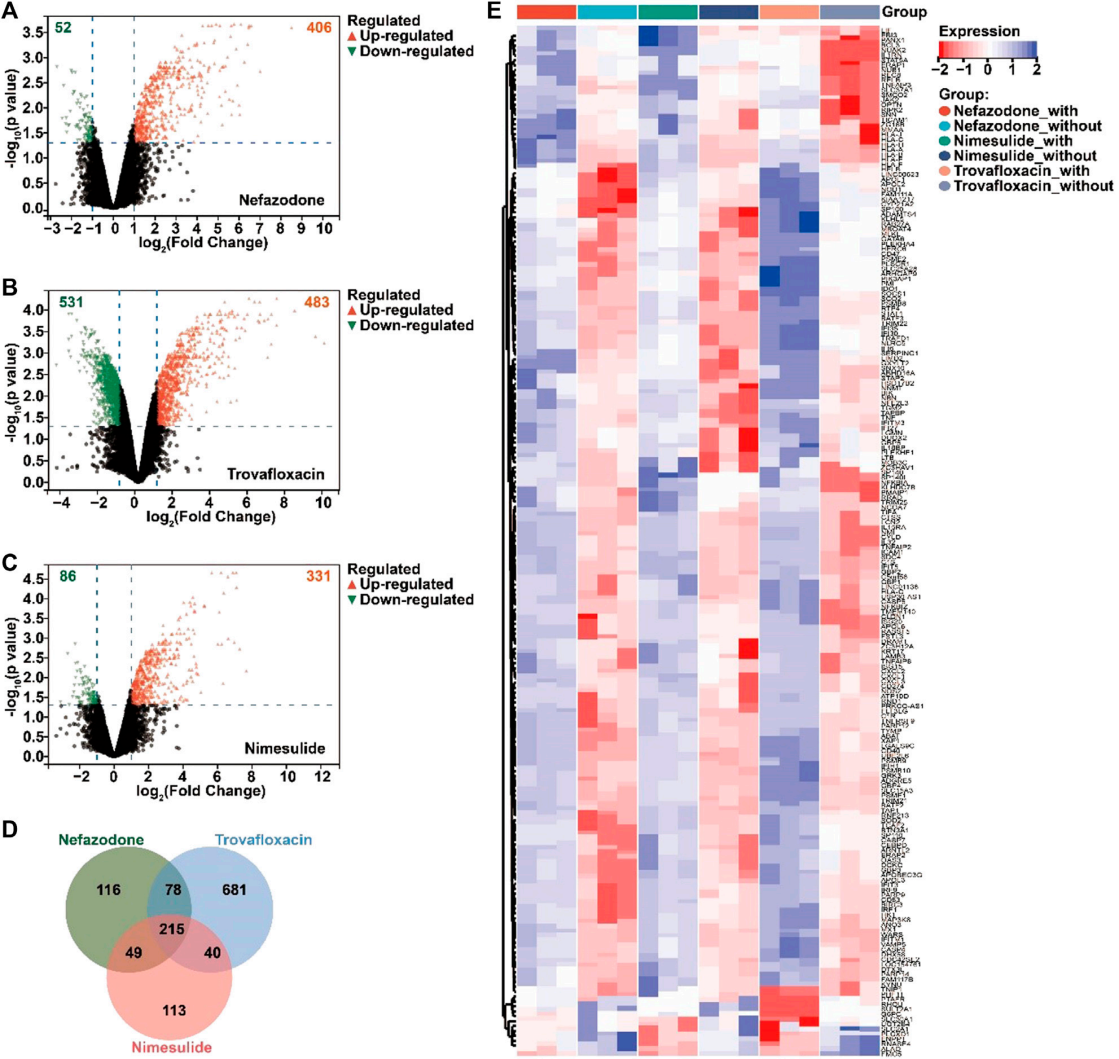
Acquired differentially expressed genes (DEGs) of DILI induced by immune inflammation. **(A–C)** Volcano diagram of the DEGs regulated by nefazodone **(A)**, trovafloxacin **(B)**, and nimesulide **(C)** under the condition of inflammatory stress in the gene expression dataset of GSE102006 (*p* < 0.05 and ǀLog_2_ fold changeǀ≥1); **(B)** the crosstalk DEGs regulated by nefazodone, trovafloxacin, and nimesulide; **(C)** heatmap of the crosstalk DEGs. Red represents upregulated DEGs and blue represents downregulated DEGs. **(D)** Venn analysis of the DEGs regulated by nefazodone, trovafloxacin, and nimesulide. **(E)** Heat map display of intersecting DEGs regulated by nefazodone, trovafloxacin, and nimesulide.

According to the GEO database, the study retrieved a gene expression dataset (numbered GSE102006) (Jiang et al., 2017) of HepG2 injury induced by three idiosyncratic drugs (nefazodone, trovafloxacin, and nimesulide) in the inflammatory microenvironment. With *p* < 0.05 and ǀLog_2_ fold changeǀ≥1 as the threshold, a total of 406 upregulated DEGs and 52 downregulated DEGs for nefazodone (Figure 2A), 483 upregulated DEGs and 531 downregulated DEGs for trovafloxacin (Figure 2B), and 331 upregulated DEGs and 86 downregulated DEGs for nimesulide (Figure 2C) were found under the stimulation of inflammatory cytokines. Among the DEGs, a total of 215 DEGs including 203 upregulated DEGs and 12 downregulated DEGs were the co-regulated DEGs among the three idiosyncratic drugs (Figure 2D) with a similar change trend (Figure 2E).

### 3.2 DILI risk inquiry of approved drugs

Under the condition of |CS| ≥ 0.60 and being included by the DrugBank database, 40 drugs (including 32 positively related drugs and 8 negatively correlated drugs) were screened with high similarity to the DILI-related gene expression signatures (Table 2). Surprisingly, among the 32 drugs with a positive correlation with DILI-related genes, 26 drugs (including 20 drugs included in the LiverTox database and 6 drugs reported in individual cases (Amitrano et al., 1992; Rank and Olson, 1989; Hannequin et al., 1988; Van Mouwerik et al., 1987; el Saghir and Hawkins, 1984; de Faria et al., 2015)) were reported to have hepatotoxicity, accounting for 81.25%, while among the 8 drugs with a negative correlation with DILI-related genes, only 3 drugs included in the LiverTox database were reported to have hepatotoxicity, accounting for 37.50%. It was suggested that the above 215 DEGs could partially predict the occurrence of DILI, and this gene set was named the DILI predictive gene set (DILI_PGS).

**TABLE 2:** 40 approved drugs with high similarity to the DILI_PGS expression signatures with |CS| ≥ 0.60.

Compound	Indication	CS	Hepatotoxicity risk
mifepristone	Cushing’s syndrome; diabetes mellitus; terminal pregnancy	0.71	Yes
ingenol mebutate	actinic keratosis	0.66	No
acetohexamide	diabetes mellitus	0.66	Yes^*^
toremifene	metastatic breast cancer	0.66	Yes
fluconazole	fungal infections	0.64	Yes
secnidazole	trichomoniasis; bacterial vaginosis	0.64	Yes
tamoxifen	metastatic breast cancer	0.63	Yes
nelfinavir	HIV	0.63	Yes
tolfenamic acid	migraine	0.63	Yes^*^
amsacrine	acute myeloid leukemia	0.63	Yes*
pergolide	Parkinson’s disease	0.62	Yes
enoxacin	gonorrhea; urinary tract infections	0.62	Yes^*^
diltiazem	hypertension; angina	0.62	Yes
clozapine	schizophrenia	0.62	Yes
rucaparib	recurrent epithelial ovarian cancer; fallopian tube cancer; primary peritoneal cancer	0.61	Yes
alectinib	non-small cell lung cancer	0.61	Yes
sirolimus	renal transplant	0.61	Yes
pirenzepine	peptic ulcer; gastric ulcer; duodenal ulcer	0.61	No
isoxsuprine	thromboangiitis obliterans; cerebrovascular insufficiency	0.61	No
cyclopenthiazide	hypertension	0.61	No
carbetocin	postpartum hemorrhage	0.61	No
fluvoxamine	depression; obsessive–compulsive disorder	0.61	Yes
fluphenazine	psychosis	0.61	Yes
aclidinium	chronic bronchitis; emphysema	0.61	Yes
vincristine	acute lymphocytic leukemia; Hodgkin lymphoma	0.60	Yes^*^
triflupromazine	psychosis; nausea; vomiting	0.60	Yes^*^
dipyridamole	postoperative thromboembolism; angina	0.60	Yes
protriptyline	depression	0.60	Yes
irinotecan	metastatic colorectal cancer	0.60	Yes
doxorubicin	disseminated neoplastic conditions	0.60	Yes
mannitol	cerebral edema	0.60	No
piperacillin	polymicrobial infections	0.60	Yes
cabozantinib	medullary thyroid cancer; renal cell carcinoma; hepatocellular carcinoma	−0.60	Yes
rocuronium	general anesthesia	−0.61	No
etomidate	general anesthesia	−0.62	No
zileuton	asthma	−0.62	Yes
guanethidine	hypertension	−0.63	No
estriol	vaginitis; vulvar itching	−0.64	No
doconexent	nutritional supplements	−0.65	No
fostamatinib	chronic immune thrombocytopenia	−0.64	Yes

Note: The *representing the evidence of Hepatotoxicity risk corresponding drugs comes from literature reports.

### 3.3 Composition of infiltrated immune cells signature for the DILI_PGS

**FIGURE 3:**
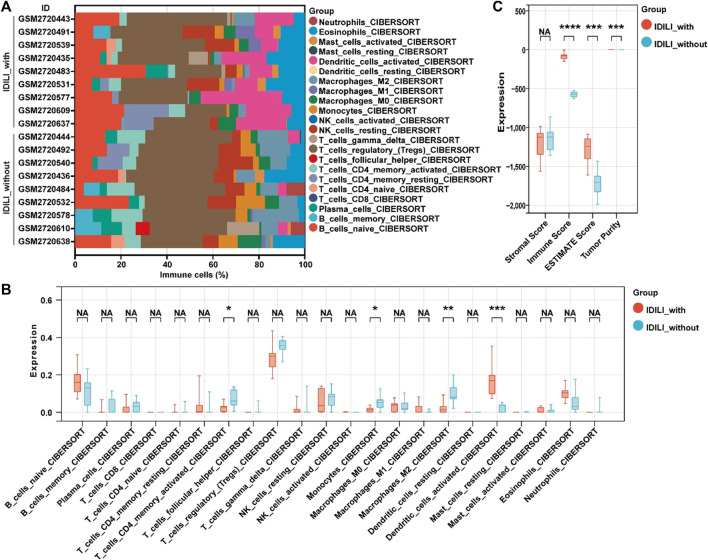
Estimation of the immune cell infiltration characterization for the occurrence of DILI induced by immune inflammation. **(A)** Stacked histogram for the relative proportion of 22 tumor-infiltrating immune cells; **(B)** the box plot of the relative proportion of 22 tumor-infiltrating immune cells estimated by CIBERSORT; **(C)** the box plot of the ESTIMATE score, immune score, stromal score, and tumor purity estimated by ESTIMATE algorithm. *p < 0.05; **p < 0.01; ***p < 0.001; NA, not significant.

To determine the relevance between the DILI_PGS risk signature and the DILI microenvironment, the CIBERSORT algorithm was used to estimate the difference of 22 types of infiltrating immune cells between the IDILI without inflammatory infiltration (IDILI_without) group and the IDILI with inflammatory infiltration (IDILI_with) group. As shown in Figure 3A, the fraction of immune cells varied significantly among the samples and groups. Meanwhile, compared with the IDILI_without group, the IDILI_with group generally contained a higher fraction of activated dendritic cells (*p* < 0.001) and a lower fraction of CD4 memory-activated T cells, monocytes, and M2 macrophages (all *p* < 0.05) (Figure 3B). In addition, the ESTIMATE algorithm was applied to calculate the ESTIMATE score, immune score, stromal score, and tumor purity, representing the HepG2 cells environment, and the result showed immune score, stromal score, and tumor purity were significantly increased in the IDILI_with group (all *p* < 0.001) (Figure 3C).

### 3.4 GSEA and DO analysis of the DILI_PGS

**FIGURE 4:**
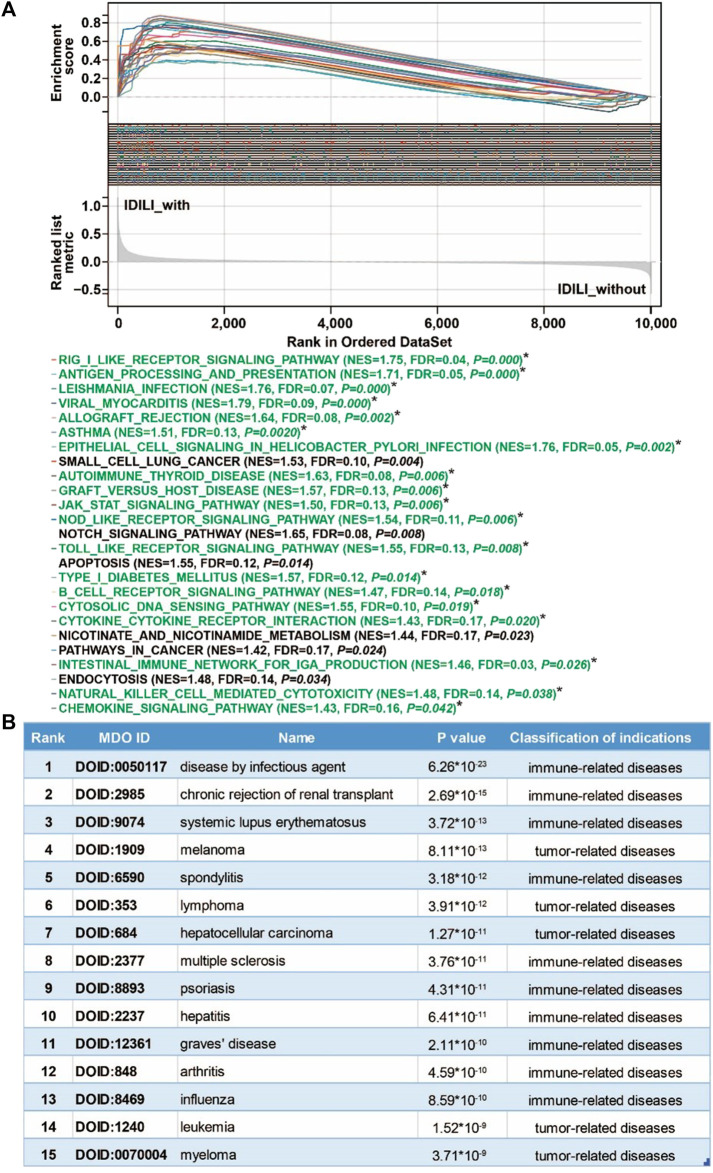
Pathways enrichment function analysis and susceptible disease characteristics analysis of the DILI predictive gene set (DILI_PGS). **(A)** GSEA analysis of the DILI_PGS (pathways marked with * in green font are immune-related); **(B)** the top 15 disease characteristics acquired using DO analysis of the DILI_PGS.

To explore the possible pathways and gene sets associated with immune functions, all expression data were divided into the IDILI_with group and IDILI_without group and then subjected to GSEA analysis. Compared with the IDILI_without group, of the 25 pathways screened according to the conditions of *p* < 0.05 and FDR <0.25 (Figure 4A), 19 pathways were highly related to immune cells and immune-related functions, including the RIG-I-like receptor signaling pathway, leishmania infection, autoimmune thyroid disease, graft-versus-host disease, the toll-like receptor signaling pathway, B cell receptor signaling pathway, cytokine-cytokine receptor interaction, natural killer cell-mediated cytotoxicity, and the chemokine signaling pathway. The details of the pathways are shown in the green font section of Figure 4A.

Based on the DO analysis via the Coexpedia platform, the DILI_PGS are mainly enriched in 300 significant disease entries (*p* < 0.05), and the top 15 disease entries were screened for display according to the *p* value. As shown in Figure 4B, the potential indications for the DILI_PGS include 10 immune-related diseases (disease by infectious agent, chronic rejection of renal transplant, systemic lupus erythematosus, spondylitis, multiple sclerosis, psoriasis, hepatitis, Graves’ disease, arthritis, and influenza) and five tumor-related diseases (melanoma, lymphoma, hepatocellular carcinoma, leukemia, and myeloma). Notably, integrating analysis of the above top 15 clinical indications curated using DO analysis and the top 25 pathways analyzed using GSEA, the results revealed that the potential indications of the DILI_PGS are primarily related to immune-related diseases. Therefore, animal models of IS (potential positive correlation) and IP (potential negative correlation) were selected as representative indications to confirm the feasibility of the DILI_PGS.

### 3.5 Screening of potential DILI risk components in BGZ

In the study, Morgan fingerprints were used to analyze the structural similarity between the components of BGZ-related monomer compounds and the three idiosyncratic drugs (nefazodone, trovafloxacin, and nimesulide) in the GSE102006 dataset for the potential hepatotoxic components in BGZ. A total of 60 components of BGZ-related monomers were obtained from the databases of TCMSP, ETCM, and the literature search, and among them, psoralen, bakuchin, and 3-Hydroxybakuchiol have high fingerprint similarity scores (all scores≥0.60) compared with the three idiosyncratic drugs (nefazodone, trovafloxacin, and nimesulide) using Morgan molecular fingerprint similarity (Table 3). It is noteworthy that psoralen has been recorded as a hepatotoxicity drug on the LiverTox platform. Therefore, psoralen was used to verify the feasibility of the DILI_PGS in predicting disease characteristics.

**TABLE 3:** The fingerprint similarity score of monomer components in BGZ.

PUBCHEM ID	Compound name	Fingerprint similarity scores			PUBCHEM ID	Compound name	Fingerprint similarity scores		
		Trovafloxacin	Nimesulide	Nefazodone			Trovafloxacin	Nimesulide	Nefazodone
643007	Xanthoangelol	0.61	0.44	0.56	15767841	Cyclobakuchiol B	0.53	0.44	0.52
5281814	Wighteone	0.55	0.56	0.47	10083762	Cyclobakuchiol A	0.53	0.44	0.52
14630492	Sophoracoumestan A	0.54	0.57	0.49	44257227	Corylinal	0.50	0.53	0.43
10606	Pyranocoumarin	0.64	0.58	0.54	5316097	Corylin	0.55	0.56	0.49
5281806	Psoralidin	0.49	0.52	0.44	5470819	4-(3,3-Dimethyl-1,4-pentadienyl)phenol	0.46	0.45	0.46
11508879	Psoralenoside	0.57	0.58	0.50	91886689	Corylifol C	0.58	0.57	0.47
5320772	Psoralenol	0.58	0.58	0.50	25056407	Corylifol A	0.54	0.54	0.45
**6199**	Psoralen*	**0.65**	**0.60**	**0.64**	5316096	Corylidin	0.62	0.58	0.52
11694710	Psoracorylifol E	0.47	0.49	0.45	11325738	Brosimacutin G	0.60	0.56	0.51
11623191	Psoracorylifol D	0.47	0.49	0.45	5280373	Biochanin A	0.48	0.54	0.44
11701986	Psoracorylifol C	0.56	0.56	0.47	5321820	Bavacoumestan B	0.57	0.57	0.50
101401747	Psoracorylifol B	0.56	0.56	0.47	5321811	Bavacoumestan A	0.58	0.57	0.50
11565494	Psoracorylifol A	0.49	0.52	0.42	5321800	Bavachromene	0.47	0.48	0.44
6476085	Psorachalcone A	0.45	0.47	0.40	5321790	Bavachromanol	0.53	0.53	0.46
5320053	Neobavaisoflavone	0.52	0.54	0.44	10337211	Bavachinin	0.51	0.52	0.47
5320052	Neobavachalcone	0.59	0.40	0.56	14236566	Bavachin	0.51	0.52	0.46
5494866	Isowighteone	0.55	0.55	0.47	6450879	Bavachalcone	0.57	0.40	0.52
11574162	Isopsoralenoside	0.58	0.58	0.51	5468522	Bakuchiol	0.59	0.57	0.58
10658	Angelicin	0.46	0.38	0.46	**3083848**	Bakuchicin*	**0.68**	**0.62**	**0.67**
101618101	Isoneobavaisoflavone	0.56	0.56	0.47	6476086	Bakuchalcone	0.53	0.53	0.46
5318608	Isoneobavachalcone	0.60	0.41	0.56	5280443	Apigenin	0.47	0.53	0.40
5889042	Isobavachromene	0.48	0.49	0.45	15818782	12,13-Epoxybakuchiol	0.46	0.38	0.43
193679	Isobavachin	0.53	0.52	0.47	14841119	8-Prenyldaidzein	0.56	0.55	0.46
5281255	Isobavachalcone	0.59	0.42	0.54	4114	Methoxsalen	0.59	0.47	0.57
5280961	Genistein	0.47	0.54	0.41	155094	6-Prenylnaringenin	0.53	0.52	0.47
10381321	Erythrinin A	0.57	0.58	0.49	2355	5-Methoxypsoralen	0.59	0.46	0.57
15818784	Delta3,2-Hydroxylbakuchiol	0.60	0.55	0.60	5321765	4′-O-Methylbroussochalcone B	0.62	0.42	0.57
128853	Delphinidin	0.46	0.54	0.40	77793	4′-Methoxyflavone	0.63	0.51	0.58
5281708	Daidzein	0.64	0.52	0.57	**56833075**	3-Hydroxybakuchiol*	**0.65**	**0.60**	**0.65**
101956380	Cyclobakuchiol C	0.57	0.46	0.55	134715107	Furano (2'',3'',7,6)-4′-hydroxyflavanone	0.54	0.56	0.49

Note: Bold font is the component with higher fingerprint similarity scores is not less than 0.60.

### 3.6 Experimental verification

#### 3.6.1 Different responses of psoralen to liver function and hepatic histological change in IP and IS animal models

**FIGURE 5:**
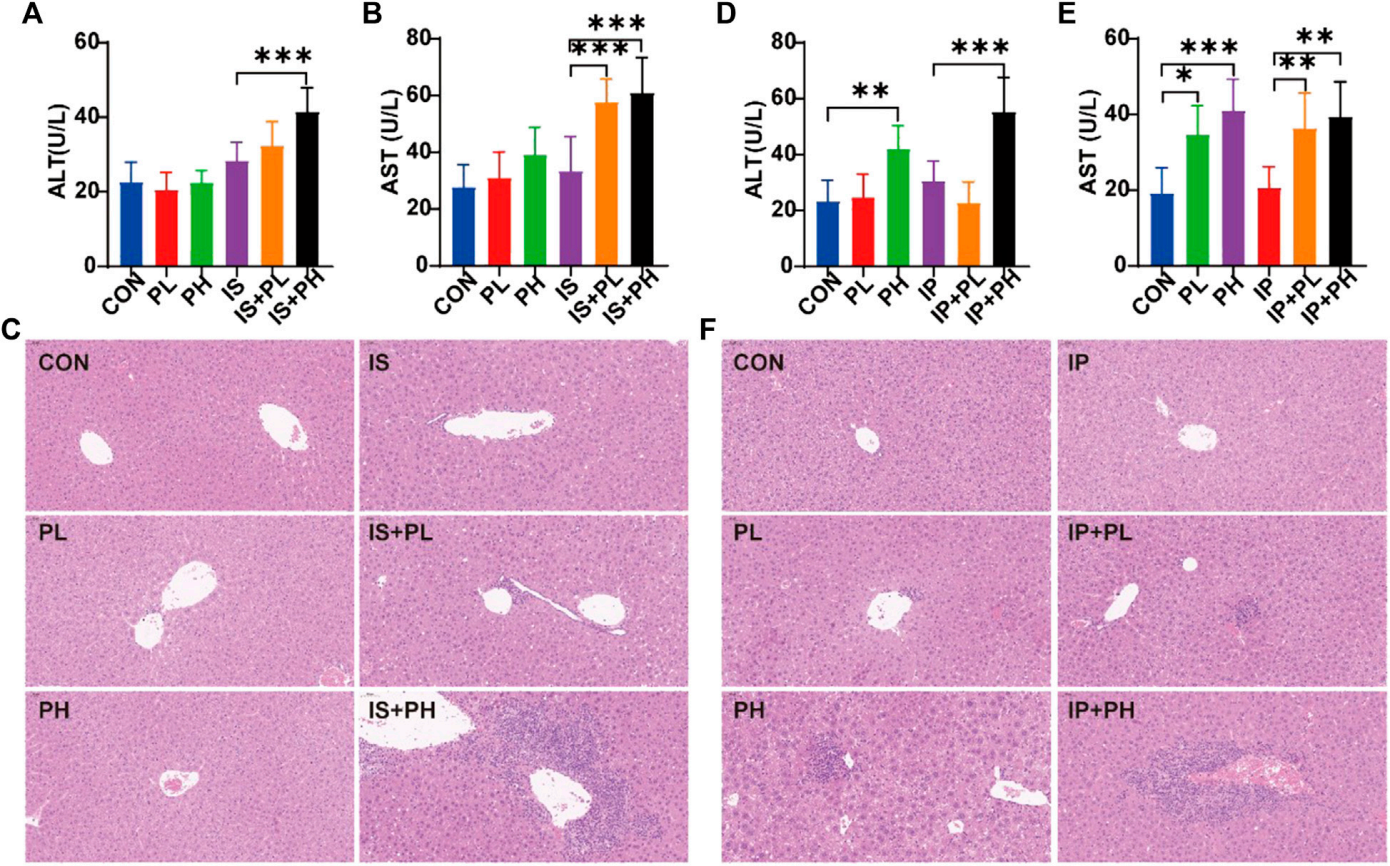
Different effects of psoralen on the liver function of IS and IP model mice. The serum levels of ALT **(A)** and AST **(B)** and the H&E staining for pathological changes of the liver **(C)** in the IS model mice. The serum levels of ALT **(D)** and AST **(E)** and the H&E staining for pathological changes of the liver **(F)** in the IS model mice. *p < 0.05; **p < 0.01; ***p < 0.001.

In the IS model, as shown in Figure 5A, compared with the CON group, the expression of ALT in PL, PH, and IS groups did not change obviously, while compared with the IS group, it was significantly increased in the IS+PH group (*p* < 0.05) with no obvious change in the IS+PL group. As for AST expression (Figure 5B), the PL, PH, and IS groups had no obvious changes compared with the CON group, while it was significantly increased in both the IS+PL and IS+PH groups compared with the IS group (both *p* < 0.05), indicating psoralen may induce liver injury when the body is under the status of immune stress; the H&E staining for pathological changes of the liver also confirmed this phenomenon (Figure 5C).

In the IP model, as shown in Figure 5D, compared with the CON group, the expression of ALT in the PL and IP groups had no obvious changes, while that in the PH group increased significantly (*p* < 0.05). Although compared with the IP group, the level of ALT in the IP+PH group increased significantly (*p* < 0.05), no significant change was seen between the IP+PH group and the PH group. As for AST expression (Figure 5E), compared with the CON group, the expression of AST in the PL and PH groups were both significantly increased (both *p* < 0.05) with no obvious change in the IP group. Although compared with the IP group, the expression of AST in the IP+PL and IP+PH groups were significantly increased (both *p* < 0.05), no significant changes were seen in the IP+PL and IP+PH groups while comparing with the PL and PH groups, respectively, indicating psoralen has certain liver accumulation toxicity, which is consistent with the literature report (Yang et al., 2020), and the pathological changes stained by H&E also confirmed this phenomenon (Figure 5F). Combined with the different responses of psoralen to the liver function of IS and IP model mice, this suggests that the immune stress status may be the potential host factor of psoralen-induced liver injury, but not the immune suppression status.

#### 3.6.2 PPI network analysis and the potential core targets of the DILI_PGS regulated by psoralen in the IS model mice

**FIGURE 6:**
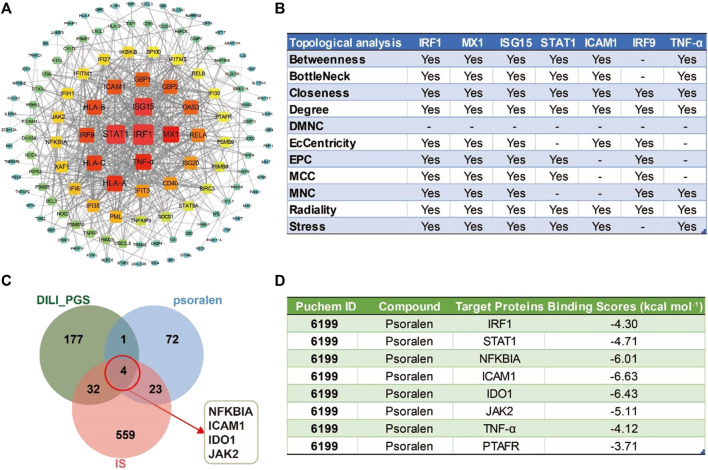
The potential core targets regulated by psoralen in IS model mice. **(A)** The PPI network for the DILI_PGS. **(B)** The seven core targets were obtained by 11 different topological analysis methods with CytoHubba. **(C)** Venn diagram of the crosstalk targets among the targets of psoralen, IS, and the DILI_PGS. **(D)** Docking scores of psoralen with the core targets.

The combined results of the PPI network analysis and the core target screening by CytoHubba for the DILI_ PGS (Figure 6A) found IRF1, MX dynamin-like GTPase 1 (MX1), interferon regulatory factor 9 (IRF9), interferon-stimulated gene 15 (ISG15), ICAM1, STAT1, and tumor necrosis factor (TNF-α) were the seven core targets with a frequency of no less than 7 calculated by 11 different topological analysis methods (Figure 6B). NFKBIA, ICAM1, IDO1, and JAK2 were the crosstalk targets among the targets of psoralen, IS, and the DILI_PG (Figure 6C).

#### 3.6.3 Molecular docking analysis of binding energy between psoralen and potential core targets

**FIGURE 7:**
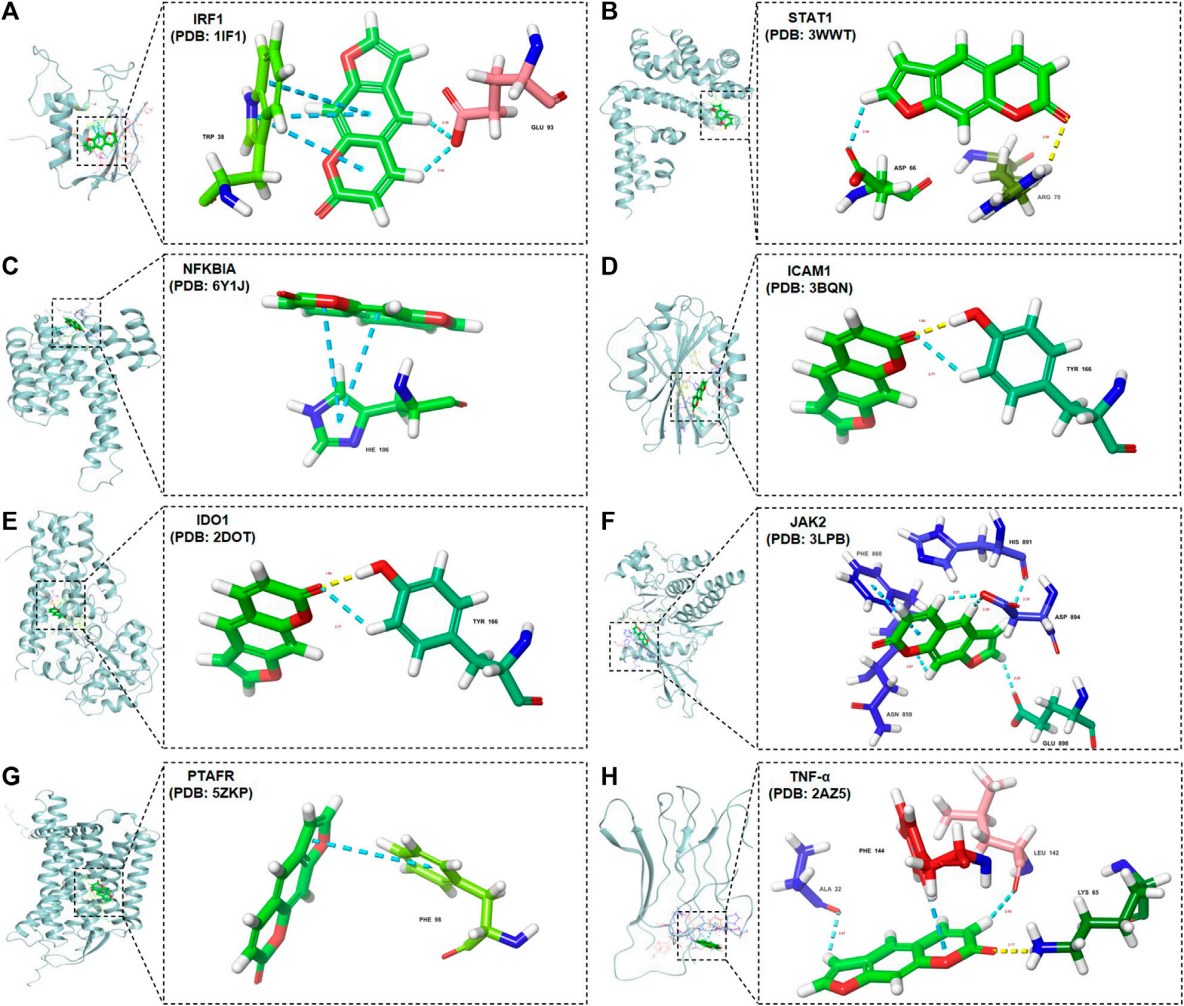
Molecular docking diagrams of core targets with psoralen. Psoralen binds to the protein **(A)** IRF1 (1IF1), **(B)** STAT1 (3WWT), **(C)** NFKBIA (6Y1J), **(D)** ICAM1 (3BQN), **(E)** IDO1 (2D0T), **(F)** JAK2 (3LPB), **(G)** TNF-α (2AZ5), and **(H)** PTAFR (5ZKP).

The intersection targets (NFKBIA, ICAM1, IDO1, JAK2, and PTAFR) between psoralen and the DILI_PGS, and the core targets (IRF1, TNF-α, and STAT1) acquired by CytoHubb, mainly acted on the TNF signal pathway (So and Ishii, 2019) and play a key role in the immune response of the body. Thus, the above targets were selected as docking proteins, and psoralen was selected as the ligand for molecular docking. The results showed that psoralen was bound to the above proteins through visible hydrogen bonds and strong electrostatic interactions, and had low binding energy of −4.300 (IRF1, PDB: 1IF1), −4.711 (STAT1, PDB: 3WWT), −6.011 (NFKBIA, PDB: 6Y1J), −6.631 (ICAM1, PDB: 3BQN), −6.431 (IDO1, PDB: 2D0T), −5.107 (JAK2, PDB: 3LPB), −4.115 (TNF-α, PDB: 2AZ5), and −3.708 (PTAFR, PDB: 5ZKP) kcal mol^-1^ (Figure 6D). Furthermore, as shown in Figures 7A–H, psoralen could easily enter and bind to the active pocket of the above proteins, indicating highly stable binding for the complexes. Thus, the above proteins may be the potential proteins regulated by psoralen, especially IDO1, ICAM1, NFKBIA, and JAK2.

#### 3.6.4 Molecular dynamics simulation of the interaction between psoralen and potential core targets

##### 3.6.4.1 Conformational stability

**FIGURE 8:**
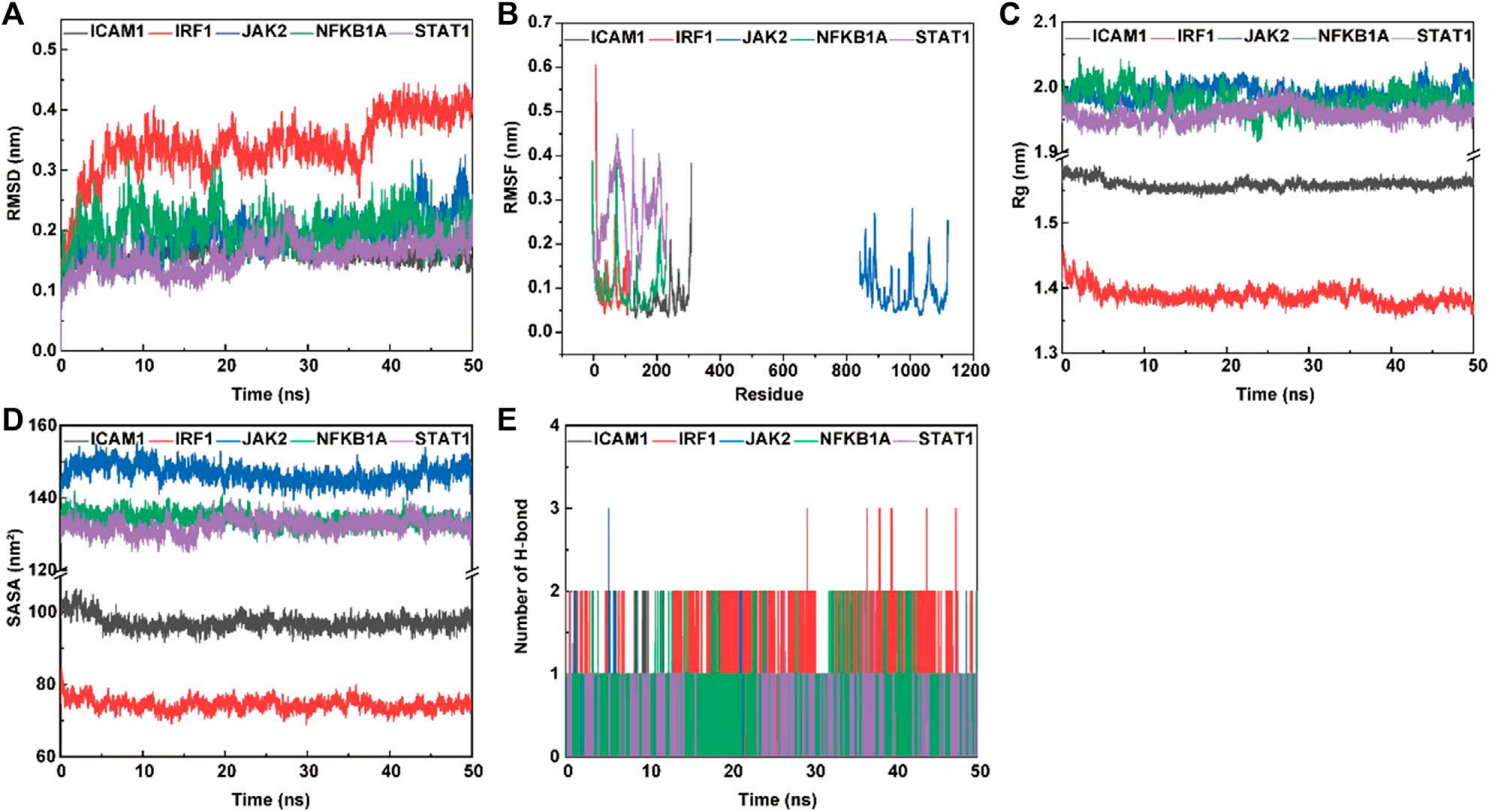
The molecular dynamics (MD) simulation of the complexes of ligand (psoralen) with proteins (ICAM1, IRF1, JAK2, NFKBIA, and STAT1). Root mean square deviation (RMSD) value **(A)**, root mean square fluctuation (RMSF) value **(B)**, radius of gyration (Rg) values **(C)**, solvent accessible surface area (SASA) **(D)**, and hydrogen bond numbers **(E)** of the complex during MD simulation.

The root mean square deviation (RMSD) and root mean square fluctuation (RMSF) plots against simulation time, shown in Figure 8, elucidated smaller fluctuations for all complexes (psoralen/ICAM1, psoralen/IRF1, psoralen/JAK2, psoralen/NFKBIA, and psoralen/STAT1) after 40 ns of the trajectory, indicating attainment of a stable conformation, and the average RMSD values of the above complexes were 0.15 nm, 0.40 nm, 0.23 nm, 0.20 nm, and 0.18 nm, respectively, while the average RMSF values of the above complexes were 0.07 nm, 0.11 nm, 0.09 nm, 0.11 nm, and 0.27 nm, respectively. The lower RMSD and RMSF values of protein–ligand docking demonstrated the structural stabilization and flexibility of the above complexes (Figures 8A, B).

##### 3.6.4.2 Compactness analysis

The radius of gyration (Rg) is employed to measure the structural variations (compactness manner and flexibility) of protein–ligand complexes during MD simulations, and the lower value of Rg describes a more rigid structure (namely, a stronger interaction between protein and ligand) in the MD simulation process (Jiang et al., 2019). The plots of Rg versus time (Figure 8C) were quite different for ICAM1 (black), IRF1 (red) JAK2 (blue), NFKB1A (green), and STAT1 (purple) with the mean values at 1.56 nm, 1.38 nm, 1.99 nm, 1.98 nm, and 1.96 nm, respectively.

##### 3.6.4.3 Solvent accessible surface area

Protein solvent accessible surface area (SASA) has been considered a critical element in the study of protein folding and stability, and a lower SASA value indicates greater compactness (Ali et al., 2014). The SASA values are quite different in range for ICAM1 (97.17 nm^2^, green), IRF1 (73.94 nm^2^, green), JAK2 (146.75 nm^2^, green), NFKB1A (133.68 nm^2^, green), and STAT1 (132.89 nm^2^, purple), respectively. This observation indicates a similar solvent interaction and also validates the compactness analysis results (Figure 8D).

##### 3.6.4.4 Analysis of hydrogen bonding

Hydrogen bond formation between the ligand and protein shows the binding stability of the complex. The simulation results showed a large number of intermolecular hydrogen bonds formed between protein residues and the ligand (psoralen), indicating a strong interaction (Figure 8E). Figure 8E shows the number of hydrogen bonds formed during the simulations. The average number of hydrogen bonds for the complexes (psoralen/ICAM1, psoralen/IRF1, psoralen/JAK2, psoralen/NFKBIA, and psoralen/STAT1) were 0.32, 1.28, 0.16, 0.77, and 0.06, respectively. The docking studies showed that psoralen formed one hydrogen bond with protease residues Tyr166 in ICAM1 and protease residues Arg70 in STAT1, respectively.

##### 3.6.4.5 Binding free energy and residue interaction energy

Molecular mechanics Poisson–Boltzmann surface area (MM/PBSA) is widely used for free energy calculation based on the MD simulation of complexes (Wang et al., 2019). The binding free energy of all complexes was calculated using the MM/PBSA method from 50 ns extracted at equal intervals of the last 10 ns MD trajectories. The contributions of different interactions were either positive or negative to the binding free energy, as summarized in Table 4 for all complexes. The binding energy of the complexes (psoralen/ICAM1, psoralen/IRF1, psoralen/JAK2, psoralen/NFKBIA, and psoralen/STAT1) were −76.215, −15.363, −9.808, −3.224, and −18.293 kJ/mol, respectively. The binding energy results showed that the psoralen/ICAM1 exhibited more stability than other complexes. Decomposition into separate energy terms revealed that polar solvation energy and comparative enthalpy variation (∆TS) decrease the binding strength of inhibitors to the protease significantly, thereby reducing the total binding energy in both complexes due to the positive energy contributions. Among the various interactions, van der Waals energy showed the most favorable contributions towards the negative binding free energy of both complexes.

**TABLE 4:** The binding free energy component of a ligand (psoralen) with proteins (ICAM1, IRF1, JAK2, NFKBIA, and STAT1) based on molecular mechanics Poisson–Boltzmann surface area (MM/PBSA) calculations.

Complex	Psoralen/ICAM1	Psoralen/IRF1	Psoralen/JAK2	Psoralen/NFKBIA	Psoralen/STAT1
Van der Waals energy (KJ/mol)	−113.514	−73.894	−29.24	−88.5	−57.052
Electrostatic energy (kJ/mol)	−8.883	−18.508	−4.981	−18.019	−23.781
Polar solvation energy (KJ/mol)	54.216	76.454	18.86	62.954	47.354
SASA energy (KJ/mol)	−14.918	−12.612	−7.48	−13.032	−11.293
Total binding energy (KJ/mol)	−83.099	−28.56	−22.842	−56.597	−44.772
∆TS^a^ (KJ/mol)	6.884	13.197	13.034	53.373	26.479
Total binding free energy (KJ/mol)	−76.215	−15.363	−9.808	−3.224	−18.293

^a^ ∆TS, refers to the entropic contribution to the free energy where T and S are the temperature and the entropy, respectively.

#### 3.6.5 Validation of potential core targets of the DILI_PGS for psoralen in IS model mice

**FIGURE 9:**
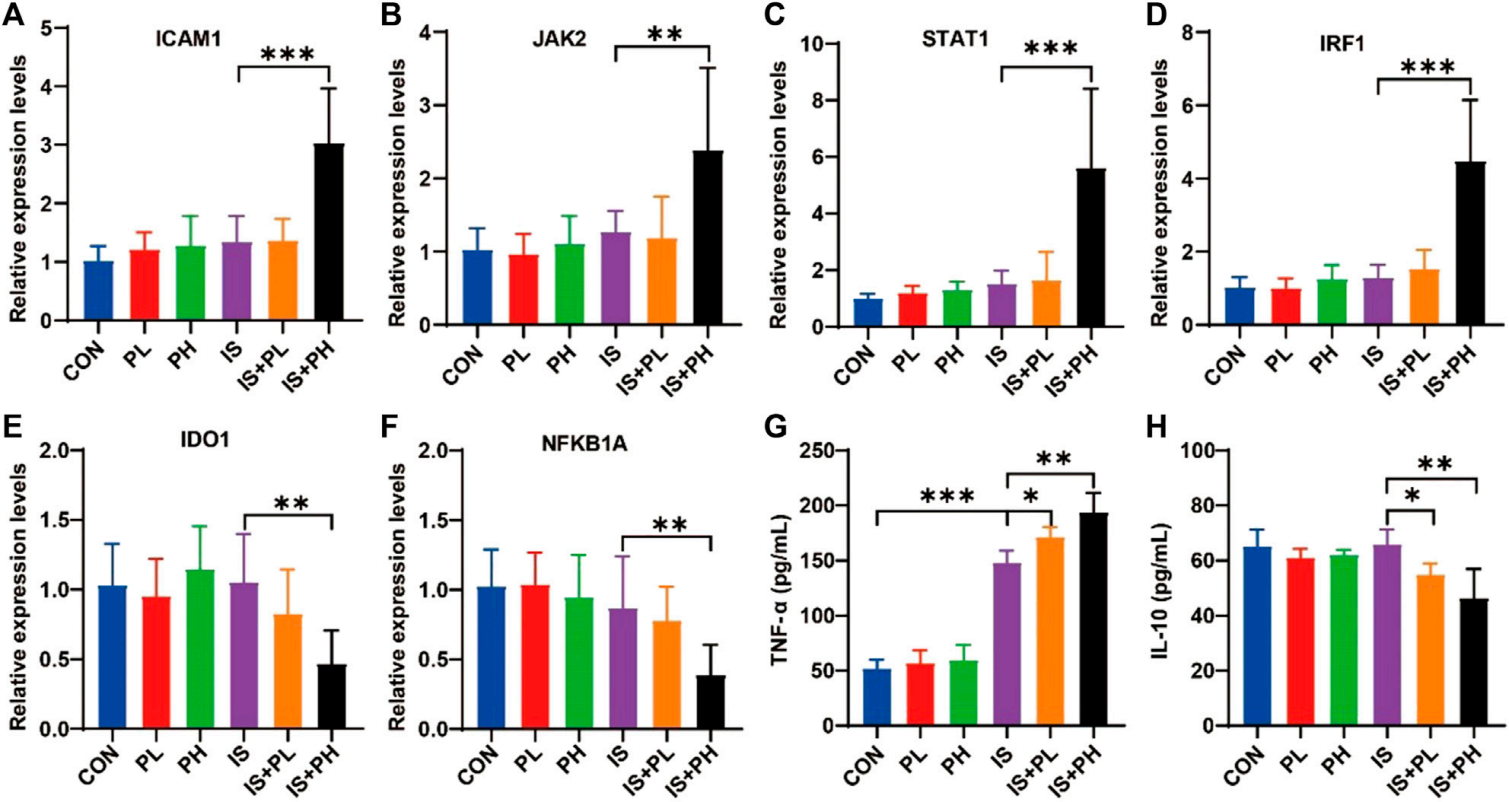
Effect of psoralen on the potential core targets expression in IS model mice. **(A–F)** The mRNA expressions of ICAM1 **(A)**, JAK2 **(B)**, STAT1 **(C)**, IRF1 **(D)**, IDO1 **(E)**, and NFKB1A **(F)** in the liver of the IS mice were checked by RT-PCR. The protein expressions of TNF-α **(G)** and IL-10 **(H)** in the liver of the IS mice were checked by ELISA. *p < 0.05; **p < 0.01; ***p < 0.001.

The results of the RT-PCR showed that psoralen significantly increased the mRNA expressions of ICAM1, JAK2, STAT1, and IRF1 (all *p* < 0.05, Figures 9A–D) and decreased the levels of IDO1 and NFKB1A in the livers of IS model mice (both *p* < 0.05, Figures 9E, F). In addition, the results of serum ELISA showed that psoralen significantly increased and decreased the protein expression of TNF-α and IL-10 in a dose-dependent manner (all *p* < 0.05, Figures 9G, H), respectively.

## 4 Discussion

In this study, a gene set that can predict the risk of DILI was constructed based on the GEO database, and 32 drugs with high similarity (positive correlation) to the DILI-related DEGs profile were found by the strategy of drug repurposing, with 81.25% of the drugs identified as having hepatotoxicity. However, only three of the eight drugs with a high similarity (negative correlation) to this gene expression profile have been reported to cause liver injury, suggesting that this gene set has a certain ability to predict the risk of DILI.

Based on this gene set, this study also predicted that the basic disease characteristics of patients inducing DILI were mainly immune-related diseases or tumor-related diseases. Among the predicted immune-related diseases, autoimmune diseases including the chronic rejection of kidney transplantation (Wojciechowski and Wiseman, 2021), systemic lupus erythematosus (Fava and Petri, 2019), spondylitis (Ward et al., 2019), multiple sclerosis (Olek, 2021), and arthritis (Burmester and Pope, 2017) are usually treated with immunosuppressants or immunomodulators, such as tacrolimus, cyclosporine, corticosteroids (hydrocortisone, prednisone, and dexamethasone), and cyclophosphamide. However, some of these immunosuppressants (including tacrolimus, cyclophosphamide, cyclosporine, and corticosteroids) were reported to have a risk of hepatotoxicity (Welz et al., 1984; Akay et al., 2006; Ko et al., 2015; Lee et al., 2022), and have been included in the LiverTox platform (https://www.ncbi.nlm.nih.gov). Hepatitis is generally considered to be the critical feature of common underlying diseases inducing DILI (Kleiner et al., 2014; Ettel et al., 2017). The immune system of patients with infectious diseases (including viral hepatitis and influenza) is usually a condition of immune-inflammatory reactions (Knolle and Thimme, 2014; Chen et al., 2018), and previous studies have shown that immune stress is a common feature of DILI (Hoofnagle and Björnsson, 2019). Among the commonly used therapeutic drugs for hyperthyroidism patients, the most obvious side effect of methimazole and propylthiouracil is hepatotoxicity. A Japanese study showed that common drugs such as propylthiouracil and metimazole in patients with Graves’ disease (hyperthyroidism) can induce the occurrence of DILI (Suzuki et al., 2019). For patients with psoriasis and psoriatic arthritis, liver injury also easily occurs when receiving the commonly used treatment drug methotrexate (Gelfand et al., 2021).

For tumor-related diseases predicted by the DILI_PGS, due to the common abnormal immune and metabolic functions in tumor patients, the immune checkpoint inhibitors (PD1 and PDL1) (Peeraphatdit et al., 2020), biosynthesis drugs that interfere with nucleic acid (methotrexate) (Ebbesen et al., 2017), drugs that affect DNA structure and function (cyclophosphamide) (Ming et al., 2019), small-molecule targeted drugs (gefitinib) (Sugiyama et al., 2015) and other commonly used clinical antitumor drugs (Oun et al., 2018) developed based on this have been widely reported to have more side effects. The liver, the most important metabolic organ of the body, has a significantly increased risk of liver injury after taking immune checkpoint inhibitors for hepatocellular carcinoma (Sangro et al., 2020).

For the above immune-related diseases or tumor-related diseases, the body generally exhibits abnormal immune activation or hypoactivation (Khan and Gerber, 2020). To verify the sensitivity of basic disease characteristic information predicted by the DILI_PGS in patients with DILI, animal models of IS and IP were used to simulate the abnormal immune activation or immunosuppressive state, respectively. As for the selection of validation drugs, psoralen, the main monomer component of BGZ which has high molecular fingerprint similarity with the IDILI drugs (nefazodone, trovafloxacin, and nimesulide) from GSE102006 was selected for follow-up study. Psoralen has been documented by the LiverTox database for hepatotoxicity. Consistent with the literature reports (Yang et al., 2020), this study found that psoralen had certain accumulative hepatotoxicity in normal mice, but, surprisingly, in the IS model mice, a single dose of psoralen only could lead to the occurrence of liver damage, while in the IP model mice, multiple doses of psoralen did not significantly increase the severity of liver damage. These results suggest that psoralen is one of the components of BGZ-induced liver injury which occurs under immune stress (Tang et al., 2022). Therefore, the immune stress state may be the potential host factor of psoralen-induced liver injury, rather than an immunosuppressive condition, and the mechanism of psoralen-induced immunogenic liver injury becomes the focus of subsequent studies.

In this study, IDO1 and ICAM1 were screened as potential crosstalk targets of psoralen regulating IS and IP via network pharmacological technology, and the results of molecular docking and molecular dynamics also revealed that psoralen has strong binding energy with IDO1 and ICAM1, respectively. IDO1 is an important immunoregulatory factor and rate-limiting enzyme of the kynurenine pathway of tryptophan metabolism, induced pro-inflammatory cytokines (IFN-γ), and other immune system mediators in response to various immune-related reactions (Merlo et al., 2020). IDO1 is associated with many disorders of immune function, including cancer, HBV infection, allergy, and autoimmune diseases (Ito et al., 2014; Liu et al., 2018; Heidari et al., 2022). Inhibition of IDO1 expression can induce immune-mediated liver injury (Affolter et al., 2019), but it has also been shown that IDO1 deficiency or inhibition can weaken acetaminophen or concanavalin A-induced liver injury (Li et al., 2021b). ICAM1, a cell surface glycoprotein and adhesion receptor, is expressed at a low basic level in immune, endothelial, and epithelial cells, but upregulated in response to inflammatory stimulation, which promotes the recruitment and activation of white blood cells from circulation to inflammatory sites (Bui et al., 2020), playing an important role in the progress of diseases including tumors, hepatitis, colitis, and cardiovascular and cerebrovascular diseases (Chen et al., 2013; Hoke et al., 2015; Zhu et al., 2015; West et al., 2017). As expected, psoralen was found to decrease the expression of IDO1 and increase the expression of ICAM1 in the liver tissue of IS model mice, suggesting that ICAM1 and IDO1 may be potential targets of psoralen-induced immunogenic liver injury.

The core proteins (IRF1, STAT1, and TNF-α) of the DILI_PGS filtered by various topological analyses in the PPI network via CytoHubba are mainly related to the activation of the TNF signaling pathway (So and Ishii, 2019), showing that TNF-α can induce the expression of IRF1 and interferon-beta (IFN-β) by regulating tumor necrosis factor receptor 1 (TNFR1) to participate in the transcriptional activation mediated by NF-κB and STAT1, inducing the increased expression of IRF1 and IFN-β and leading to the prolonged expression of pro-inflammatory chemokines via STAT1 (Yarilina et al., 2008). IRF1 can also act synergistically with STAT1 to induce the expression of IFN-stimulated genes in IFN γ-treated cells (Feng et al., 2021). The results of molecular docking and molecular dynamics in this study showed that psoralen had a good binding ability with IRF1 and STAT1, and the animal experiment further demonstrated that psoralen could significantly increase the expression of IRF1, JAK2, STAT1, and TNF-α and significantly decrease the levels of IL-10 and NFKB1A in IS model mice. The above results suggest that the occurrence of immunogenic liver injury induced by psoralen may be related to the activation of the TNF signaling pathway.

In conclusion, based on the strategy of integrating gene expression profile similarities via CMap and Coexpedia, this study constructed a gene set with high accuracy to predict the occurrence of DILI, and this gene set revealed that the immune activation status is one of the main host factor inducing DILI. The study also found and confirmed that psoralen is one of the toxic components of BGZ with immunogenic hepatotoxicity, which extends the cognitive category that psoralen only accumulates hepatotoxicity as previously reported.

## Data Availability

The original contributions presented in the study are included in the article/Supplementary Material, further inquiries can be directed to the corresponding author.
