# Supplementary material for: A gene expression profile-based approach to screen the occurrence and predisposed host characteristics of drug-induced liver injury: a case study of Psoralea corylifolia Linn
Source: Front Chem. 2023 Oct 6;11:1259569. doi: 10.3389/fchem.2023.1259569 (PMC10588485; doi:10.3389/fchem.2023.1259569)
Supplement: Supplementary file 1 [file Table1.docx]

**Supplementary Table**

**Supplementary Table 1.** The online resources used in this study.

| **Number** | **Website** | **Abbreviation** | **Function Introduction** |
| --- | --- | --- | --- |
| 1 | https://www.ncbi.nlm.nih.go | LiverTox | LiverTox® provides up-to-date, unbiased, and easily accessed information on the diagnosis, cause, frequency, clinical patterns, and management of liver injury attributable to prescription and nonprescription medications and selected herbal and dietary supplements |
| 2 | https://www.fda.gov/science-research/liver-toxicity-knowledge-base-ltkb/drug-induced-liver-injury-rank-dilirank-dataset | DILIrank | The DILIrank dataset consists of 1,036 FDA-approved drugs |
| 3 | https://clue.io/query | CMap | A database platform that contains more than 5,000 marketed drugs or small molecule compounds with potential pharmacological activity to interfere with the expression of perturbed genes in 80 human cell lines and provides a method to quantify the disease-drug similarity "link score" criteria, realizing the perfect combination of computer technology and high-throughput omics database |
| 4 | https://www.coexpedia.org/ | Coexpedia | Coexpedia is a database of context-associated co-expression networks inferred from individual series of microarray samples for human and mouse of GEO. |
| 5 | https://www.ncbi.nlm.nih.gov/geo/ | GEO | GEO is a public functional genomics data repository for users to upload, query, and download experiments and curated gene expression profiles. |
| 6 | https://go.drugbank.com/ | Drugbank | DrugBank database is a bioinformatics and chemoinformatics database. It combines detailed drug data with comprehensive drug target information. |
| 7 | https://cibersort.stanford.edu/ | CIBERSORT | CIBERSORT is an analytical tool from the Alizadeh Lab and Newman Lab to impute gene expression profiles and provide an estimation of the abundances of member cell types in a mixed cell population, using gene expression data. |
| 8 | https://bioinformatics.mdanderson.org/estimate/ | ESTIMATE | ESTIMATE provides researchers with scores for tumor purity, the level of stromal cells present, and the infiltration level of immune cells in tumor tissues based on expression data. |
| 9 | http://sangerbox.com/home.html | Sangerbox | Sangerbox is an online website capable of conducting bioinformatics analysis and bioinformatics mapping. |
| 10 | http://www.gseamsigdb.org/gsea/downloads.jsp | MSigDB | The Molecular Signatures Database (MSigDB) is a collection of annotated gene sets for use with GSEA software. |
| 11 | https://tcmsp-e.com/tcmsp.php | TCMSP | TCMSP is a unique systems pharmacology platform of Chinese herbal medicines that captures the relationships between drugs, targets, and diseases. |
| 12 | http://www.tcmip.cn/ETCM/index.php/Home/Index/ | ETCM | ETCM includes comprehensive and standardized information for the commonly used herbs and formulas of TCM, as well as their ingredients. |
| 13 | https://pubchem.ncbi.nlm.nih.gov/ | PubChem | PubChem is the world's largest collection of freely accessible chemical information. |
| 14 | https://cn.string-db.org/ | STRING | STRING is a database of known and predicted protein-protein interactions. |
| 15 | https://www.pdbus.org/ | RCSB | RCSB Protein Data Bank (RCSB PDB) provides access and tools for exploration, visualization, and analysis of Experimentally-determined 3D structures from the Protein Data Bank (PDB) archive and Computed Structure Models (CSM) from AlphaFold DB and ModelArchive. |
| 16 | https://www.uniprot.org/ | Uniprot | UniProt is the world’s leading high-quality, comprehensive, and freely accessible resource of protein sequence and functional information. |
